# Residual Aneurysmal Sac Shrinkage Post-Endovascular Aneurysm Repair: The Role of Preoperative Inflammatory Markers

**DOI:** 10.3390/biomedicines11071920

**Published:** 2023-07-06

**Authors:** Edoardo Pasqui, Gianmarco de Donato, Cecilia Molino, Mustafa Abu Leil, Manfredi Giuseppe Anzaldi, Giuseppe Galzerano, Giancarlo Palasciano

**Affiliations:** Vascular Surgery Unit, Department of Medicine, Surgery and Neuroscience, University of Siena, 53100 Siena, Italy

**Keywords:** abdominal aortic aneurysm, EVAR, inflammation, vascular surgery, arterial remodeling, biomarkers

## Abstract

Introduction: In this study, we evaluated the role of preoperative inflammatory markers as Neutrophil-to-Lymphocyte (NLR) and Platelet-to-Lymphocyte (PLR) ratios in relation to post-endovascular aneurysm repair (EVAR) sac shrinkage, which is known to be an important factor for abdominal aortic aneurysm (AAA) healing. Methods: This was a single-center retrospective observational study. All patients who underwent the EVAR procedure from January 2017 to December 2020 were eligible for this study. Pre-operative blood samples of all patients admitted were used to calculate NLR and PLR. Sac shrinkage was defined as a decrease of ≥5 mm in the maximal sac diameter. The optimal NLR and PLR cut-offs for aneurysmal sac shrinkage were obtained from ROC curves. Stepwise multivariate analysis was performed in order to identify independent risk and protective factors for the absence of AAA shrinkage. Kaplan–Meier curves were used to evaluate survival rates with respect to the AAA shrinkage. Results: A total of 184 patients were finally enrolled. The mean age was 75.8 ± 8.3 years, and 85.9% were male (158/184). At a mean follow-up of 43 ± 18 months, sac shrinkage was registered in 107 patients (58.1%). No-shrinking AAA patients were more likely to be older, to have a higher level of NLR and PLR, and be an active smoker. Kaplan–Meier curves highlighted a higher rate of survival for shrinking AAA patients with respect to their counterparts (*p* < 0.03). Multivariate analysis outlined active smoking and NLR as independent risk factors for no-shrinking AAA. Conclusions: Inflammation emerged as a possible causative factor for no-shrinking AAA, playing a role in aneurysmal sac remodeling. This study revealed that inflammatory biomarkers, such as NLR and PLR, can be used as a preoperative index of AAA sac behavior after EVAR procedures.

## 1. Introduction

Abdominal aortic aneurysm (AAA) is defined as a permanent dilatation of the abdominal aorta. Its natural history is progressive growth in the majority of patients, until rupture with high rates of morbidity and mortality [1]. Pre-emptive treatment is essential to reduce the incidence of AAA rupture. In the last three decades, endovascular aneurysm repair (EVAR) has become an essential alternative to open surgical repair, gaining more and more evidence with respect to safety and effectiveness in both elective and emergent settings [1]. Recently, the behavior of the residual aneurismal sac has gained significance as a predictor of “patient healing”. In the absence of evident endoleaks, sac stability or an increase appeared to be associated with lower rates of overall mortality and reinterventions [2,3,4,5,6]. Sac shrinkage, defined as a reduction in the AAA maximum diameter >5 mm is associated with technically and clinically better outcomes [7]. Patients’ baseline characteristics, anatomical features, and type of endograft were studied as potential influencing factors on post-EVAR sac behavior with conflicting results [8]. Up to now, the mechanisms and factors underlying sac shrinkage are not well established. Inflammation theory is receiving increasing interest across a variety of medical and surgical fields as a reliable predictor of complications and worse outcomes, especially for cardiovascular disease [9,10,11]. The hypothesis is that pre-operative inflammatory status could be an influencing factor on AAA sac behavior and indirectly related to patients’ survival.

Starting from the point that the Neutrophil-to-Lymphocyte ratio (NLR) and Platelet-to-Lymphocyte ratio (PLR) are two inexpensive and reliable indexes that could be used to measure the patient systemic inflammatory status [12,13], we decided to investigate the role of NLR and PLR in predicting post-EVAR sac shrinkage and overall mortality.

## 2. Materials and Methods

### 2.1. Patients’ Selection

All patients who underwent elective EVAR procedures from January 2017 to December 2020 were considered eligible in the study. A total of 276 patients were initially included in the study; of them, 92 were excluded from the analysis because they did not meet all the inclusion criteria. Every hospitalization was retrospectively analyzed in terms of the anamnestic features, medications, preoperative blood samples (white blood count, neutrophil and lymphocyte subpopulation, and platelets), and types of procedures performed. Coronary artery disease (previous history of coronary-artery-related events with or without surgical or endovascular interventions), hypertension (blood pressure higher than the guidelines suggested cutoff value or antihypertensive drug assumption [14]), diabetes mellitus (defined as occasional plasma glucose value of ≥200 mg/dL (≥11.1 mmol/L), or fasting plasma glucose of ≥126 mg/dL ( 7.0 mmol/L) (fasting time 8–12 h), or OGTT 2-h value in venous plasma ≥200 mg/dL (≥11.1 mmol/L) or assumption of insulin or antidiabetic drugs), chronic obstructive pulmonary disease (defined as a diagnosis of chronic bronchitis and emphysema, classic asthma, or a combination of the above), renal disease (chronic renal insufficiency defined by serum creatinine >1.2 mg/dL), smoking history (any current or past regular use of tobacco), congestive heart failure, history of cerebrovascular events (stroke and/or transient ischemic attacks), history of cancer (any current or past incidence if malignancy), dyslipidemia, and atrial fibrillation were considered as comorbidities. The number of drugs taken by patients was also considered. In the post-operative period, medical charts were reviewed in order to identify procedure-related complications, in particular the contrast-induced nephropathy (CIN), defined as an absolute (≥0.5 mg/dL) or relative increase (≥25%) in serum creatinine at 48–72 h after exposure to a contrast agent compared to baseline serum creatinine values, when alternative explanations for renal impairment have been excluded [15] and post-implantation syndrome defined as the presence of fever (persisting body temperature > 38 °C lasting for more than 1 day during hospitalization) and leukocytosis (white blood cell count > 12.000/μL) with negative blood culture results. The biochemical inflammatory markers were analyzed, calculating the NLR and PLR. The NLR and PLR were obtained by dividing the absolute neutrophil and platelet counts by the lymphocyte count. The endografts used for treatment were selected by single operator’s preference based on patients’ anatomical features, endograft availability, and platform characteristics. Indication to EVAR was AAA diameter of ≥5.5 cm in males and ≥5 cm in females, or if the aneurysm was rapidly increasing (increasing diameter ≥ 0.5 cm in 6 months, or ≥1 cm in 12 months) [1]. Patients’ demographics, intraoperative data, and post-operative outcomes were collected through hospital charts. Preoperative imaging was routinely performed with an angio-computed tomography scan of the abdominal aorta of 1 mm slice thickness. Follow-up included a clinic visit assessing serum creatinine level and computed tomographic angiography at 1 month. Color-flow duplex ultrasound (DUS) or contrast-enhanced ultrasound US was performed at 6 months, 12 months, and annually thereafter to assess the stent graft, aneurysm size, and the presence of endoleaks. A computed tomographic angiography was rescheduled when the duplex US or contrast-enhanced US revealed significant endoleaks, sac enlargement >5 mm, or any suspicion of procedure-related complications. Residual aneurysmal sac shrinkage was defined as a reduction of 5 mm or more in the diameter of AAA with respect to the preoperative maximum diameter comparing the same kind of imaging, DUS, or CT. Patients were excluded from the analysis if they did not fulfil a minimum of 6 months follow-up after intervention. Patients with a known history of infection, hematological disease, pre-operative intake of steroids or immunomodulators drugs, or coronary/cerebrovascular acute events in the previous six months were also excluded from the study due to the possible influence of preoperative inflammatory markers. The ethical committee was informed of the no-experimental design of the retrospective investigation and endorsed the study. An informed consent waiver was approved by the ethical committee due to the retrospective design of this study based on patient records. We performed the study in accordance with the Declaration of Helsinki.

### 2.2. Outcomes Definition

The primary outcome was the association of the preoperative values of NLR and PLR with post-EVAR sac shrinkage. Survival of no-shrinking and shrinking AAA was evaluated as a secondary outcome. Technical success was defined on an intent-to-treat basis and required the successful introduction and deployment of the device in the absence of surgical conversion or mortality, type I or III endoleaks, or graft limb obstruction. Clinical success was defined as success without the need for an additional or secondary surgical or endovascular procedure [2].

### 2.3. Statistical Analysis

The categorical data were reported as numbers and percentages. The means (±standard deviation) were used to analyze the continuous variables. A Student’s *t*-test was used to compare the normally distributed variables. Mann–Whitney U tests were used to com- pare the non-normally distributed variables. The categorical variables were compared using a Fisher’s exact test. The statistical significance was a *p*-value of less than 0.05. An analysis of the NLR and PLR for aneurysmal sac shrinkage was conducted. The NLR and PLR were studied via receiver operating characteristic (ROC) curves with aneurysm sac shrinkage. Optimal cut-offs were chosen based on the specificity and sensitivity. Moreover, survival rates were evaluated dividing the total population between shrinking AAA and no-shrinking AAA groups using Kaplan–Meier curve method. Log-Rank test was used to compare the two survival curves. The datasets were analyzed using univariate methods with the aim of determining the independent factors correlated to no-AAA shrinkage. A Cox proportional analysis was used to determine the independent predictors for no-AAA shrinkage; a probability of 0.10 was used to enter the variables into the Cox model in a forward-stepwise manner. A probability of 0.15 was used to remove the variables from the model. Independent predictor variables that contributed to the final multivariate model were considered to be significant risk factors for restenosis if they achieved a two-sided *p* < 0.05. All statistical analyses were performed with GraphPad Prism 9.0 (GraphPad Software Inc., San Diego, CA, USA) and StatPlus Build 7.1.1 (Analysis Soft Inc., Walnut, CA, USA).

## 3. Results

A total of 184 patients were included in the study. Mean age was 75.8 ± 8.3 years, and 85.9% were male (158/184). The population study was characterized by a high prevalence of cardiovascular risk factors: 84.2% (155/184) had hypertension, 69.6% (128/184) had dyslipidemia, and 22.8% (41/184) had chronic obstructive pulmonary disease. The full details are listed in Table 1.

EVAR was performed under local/blockage anesthesia in all cases, but 25 cases (13.6%) required general anesthesia. In more than half of the cases, a procedure was performed via bilateral femoral surgical access (54.4%). The mean duration of the procedure was 123 ± 52 min.

Primary technical and clinical success was reached in 98.6% and 96.7%, respectively. Thirteen (7%) cases experienced postoperative CIN with no long-term consequences during hospitalization. Post-implantation syndrome was registered in 29.9% of cases (55/158). Only one major adverse event was registered: one patient suffered from an acute myocardial infarction, promptly treated with an endovascular revascularization. No in-hospital deaths were registered. Mean hospitalization was 5.1 ± 2.4 days. A full list of the data is highlighted in Table 2.

Mean sac shrinkage of the entire population was −4.9 ± 8.1. The number of shrinking AAAs was 107 (58.2%). The analysis between shrinking and no-shrinking AAA highlighted that no-shrinking AAAs were more likely to be older and to have an active smoking habit. In the no-shrinking AAA group, we registered 10 cases of sac enlargement. Lab testing outlined that no-shrinking AAA had a lower level of lymphocyte and higher level of NLR and PLR. The full details are listed in Table 3.

**Figure 1:**
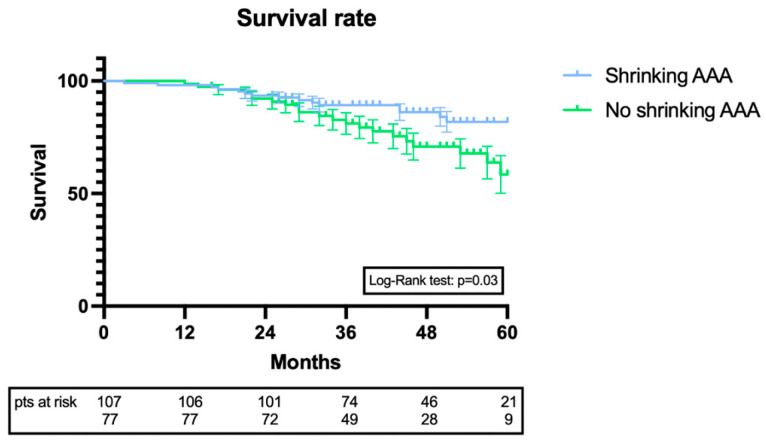
Kaplan–Meier curve comparison between shrinking and no-shrinking AAA.

During a mean follow-up of 43 ± 18 months, 37 deaths were registered. None of them were AAA-related. During follow-up, 13 endoleaks were diagnosed, 6 type II endoleaks with no need for reintervention, 4 type Ib endoleaks, and 3 type Ia endoleaks. All of the type Ia and Ib endoleaks were treated via an endovascular approach with good exclusion of the AAA. Endoleaks were more common in the no-shrinking AAA (*p* = 0.04). Kaplan–Meier analysis was performed in order to evaluate the survival rate between shrinking AAA and no-shrinking AAA. For the shrinking AAA group, the survival rate was 99.1%, 98.1%, 93.4%, 89.2%, 86.1%, and 81.8% at the 6-, 12-, 24-, 36-, 48-, and 60-month follow-up, respectively. For the no-shrinking AAA group, the survival rate was 100%, 100%, 92.1%, 81.1%, 70.8, and 58.5% at the 6-, 12-, 24-, 36-, 48-, and 60-month follow-up, respectively. The Log-Rank test revealed that the two curves were significantly statistically different (*p* = 0.03) (Figure 1).

### 3.1. Inflammatory-Biomarker-Related Outcomes

**Figure 2:**
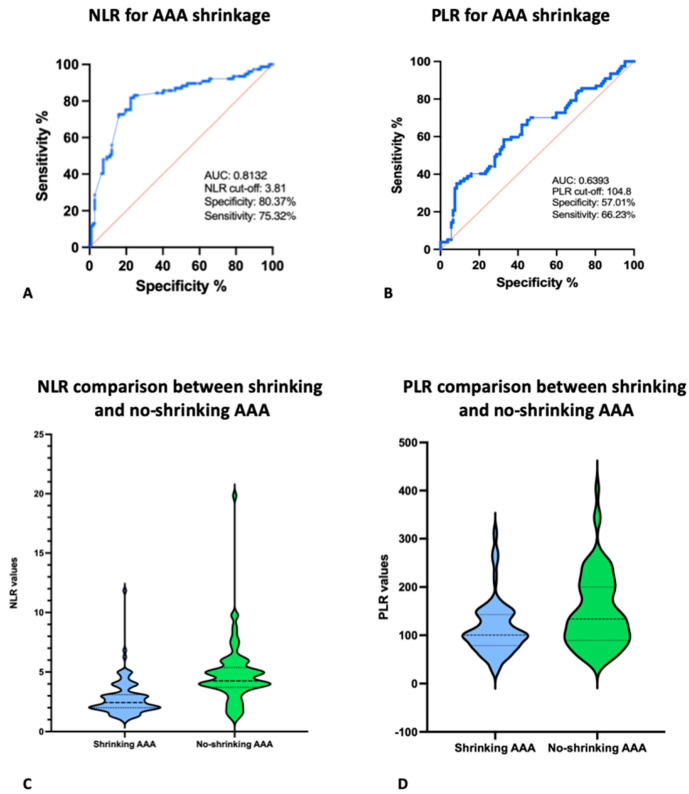
(**A**,**B**) ROC curves for optimal value of NLR and PLR for AAA shrinkage; (**C**,**D**): NLR and PLR mean values comparison between shrink and no-shrinking AAA.

The baseline laboratory data are reported in Table 1. Mean NLR was 3.6 ± 2.2, while mean PLR was 135.5 ± 73. Both NLR and PLR values were statistically different between subgroups of shrinking and no-shrinking aneurysm (*p* < 0.001). (Figure 2) Moreover, NLR and PLR were also calculated comparing patients without and with a diagnosis of endoleak. NLR was 4.2 ± 1.7 and 3.5 ± 2 for patients with and without EL, respectively (*p* = 0.2), and PLR was 139.6 ± 69 and 133.6 ± 71 (*p* = 0.3). The single cut-offs for the NLR and PLR were calculated, and ROC curves were obtained to analyze the effect of the NLR and PLR with sac shrinkage. The ROC curves identified the following values: an NLR < 3.81 was selected as the cut-off for AAA shrinkage (sensitivity of 75.32% (95% CI 64.65–83.60%) and specificity of 80.37% (95% CI 71.85–86.79%)) with an area under the curve (AUC) of 0.8132 (95% CI 0.7465–0.8799; *p* < 0.0001); a PLR < 104.8 was selected as the cut-off for AAA shrinkage (sensitivity of 66.23% (95% CI 55.12–75.8%) and specificity of 57.01% (95% CI 47.55–65.99%)) with an AUC of 0.6393 (95% CI 0.5566–0.7219; *p* = 0.0013).

### 3.2. Multivariate Analysis

The variables identified as significant from the univariate analyses were entered into the Cox regression analysis. Multivariate analysis highlighted that active smoking (HR 2.16, CI: 1.72–3.06, *p* = 0.04) and NLR value (HR 1.78, CI: 1.66–2.67, *p* = 0.02) were independent risk factors for no-shrinking AAA. The full details are listed in Table 4.

## 4. Discussion

The present study highlights that inflammation could be associated with the mechanism underlying sac shrinkage after EVAR. NLR and PLR, identified as indirect measures of patient’s inflammatory status, seem to be linked to post-EVAR sac shrinkage since higher levels of NLR and PLR values were linked to a lower rate of shrinking AAA. In addition, this paper confirms that patients who did not experience an AAA sac shrinkage > 5 mm had lower survival rates with respect to the counterpart, especially on medium–long follow-up.

In the last two decades, post-EVAR sac shrinkage has been extensively studied to understand its role as an early predictor of aneurysm healing. Persistent shrinkage has been suggested as a highly sensitive predictor of survival in EVAR patients. Cieri et al. reported that this selected group of patients reached very high survival rates: at 3 and 10 years of 100 and 99.7%, respectively [16]. Additionally, Lee at al. reported that an AAA volume reduction > 10% at 6 months follow-up after EVAR was associated with high rates of clinical success; different grades of volume reduction were associated with different rates of clinical success [17]. More recently, a large analysis from the Vascular Quality Initiative program highlighted that aneurysm sac behavior after EVAR was associated with the development of newly diagnosed endoleaks, reinterventions, and long-term mortality. The authors also reported that not only sac expansion but any failure of the sac to regress is associated with higher long-term mortality, independent of reinterventions or endoleaks [18]. In this light, the literature has proven that sac regression failure could be directly or indirectly linked to patients’ mortality.

The real mechanism and the identification of factors associated or not to sac regression are still ongoing. Anatomical features of the AAA have been discussed with conflicting results, for example, aortic neck length, angle and diameter, presence of iliac aneurysms, and inferior mesenteric artery or lumbar artery patency [19,20,21]. Preoperative AAA diameter has also been advocated as a potential factor affecting post-EVAR sac regression, with no clear evidence. Some studies have shown that large AAAs were more likely to have sac regression [22,23]. On the contrary, some experience highlighted that small-diameter AAAs regress more and have better outcomes [24].

Calcifications and intra-aneurysmal thrombus burden still remain debatable. Nakayama et al. reported that a lower extent of calcification correlated with accelerated expansion [25], while Lindholt et al. showed that, in a cohort of more than a hundred patients, AAA calcifications > 50% were associated with a higher rate of sac regression [26]. Intra-aneurysmal thrombosis burden was identified as a considerable variable in relation to aneurysmal sac remodeling, with contradictory results. A retrospective review of 100 patients revealed that thrombus burden on preoperative computed tomography angiography was a strong independent factor of sac regression following EVAR [27]. Sadek et al. confirmed this finding. An increased proportion of thrombus on pre-EVAR CT resulted in a greater likelihood of sac shrinkage; in addition, patients with enlarging AAA on post-operative CT had less sac thrombosis on pre-EVAR than patients without evidence of an endoleak [28]. On the other hand, a metanalysis conducted on 24 studies focused on the potential influencing factor on sac regression, highlighting that sac thrombus had a nearly significant negative impact on sac regression tendency [29].

All the data presented confirm that sac regression remains a “black box” in term of predisposition and risk factors due to a large amount of literature that did not convey a straightforward interpretation. Baseline patient characteristics must influence sac regression, and systemic inflammation could be an important point to focus on.

Systemic inflammation was recently linked to adverse cardiovascular and non-cardiovascular events in various fields of medical and/or surgical settings [4,30,31,32]. Specifically, cardiovascular diseases are particularly prone to this mechanism; in fact, the CANTOS study demonstrated that the use of an interleukin 1b inhibitor significantly reduced the rate of cardiovascular events by 17% [33]. AAA patients have, per se, a high risk for cardiovascular events, and patients with a high pre-operative level of inflammation due to the intrinsic nature of their aneurysmal pathology and/or comorbidities experienced different rates of mortality stratified for the behavior of their excluded aneurysmal sac. Aortic dynamic and aortic wall pressure could be a stimulating factor for the release of inflammatory markers as cytokines. Aneurysmal disease is associated with high levels of proinflammatory mediators, such as interleukin and metalloproteinase. No-shrinking aneurysms continue to release matrix metalloproteinases and cytokines [34,35].

In this perspective, shrinking or no-shrinking AAA could be an index of individual biological and clinical frailty. A recent experience published by Bradley at al. supported that the systemic inflammatory grade (SIG) obtained via NLR and the modified prognostic score predicted 1-year mortality after EVAR, and, additionally, an increased level of SIG was linearly associated with increasing mortality rates [36]. Our study highlighted that a value of NLR < 3.81 is independently associated with shrinking AAA; in other words, patients with a lower level of preoperative inflammatory status are more likely to experience a post-EVAR sac regression and consecutively higher rates of mid- and long-term survival. The literature has already outlined peculiar links between NLR- and EVAR-related outcomes. Octeau et al. confirmed that a higher NLR value was significantly associated with mortality and reinterventions following EVAR and could be considered as an independent predictor of mortality after checking for factors, such as age, AAA diameter, and clinical comorbidities [37]. These data are in line with the literature and our findings; no data were presented regarding residual sac diameter evolution after EVAR. A recent experience also proved that higher levels of NLR and PLR were also related to acute kidney injury after EVAR, in particular the immediate post-operative values with respect to the pre-operative ones [38]. This observation could imply that not only the preoperative inflammatory status could be of interest but also the trend and the modifications through the perioperative period.

### Limitations

The paper has some limitations. First, the retrospective and observational nature of the study could preclude the opportunity to derive direct cause-and-effect risk associations; second, study population was limited in terms of size; third, it was impossible to take into account other inflammatory markers (such as C-reactive protein) that are not usually evaluated before the execution of this type of surgical procedure.

## 5. Conclusions

Aneurysm sac regression is becoming an interesting marker of patient healing. The mechanism underlying the post-EVAR sac remodeling is not fully understood. Inflammation has emerged as a possible causative factor for no-shrinking AAA, playing a role in aneurysmal sac remodeling. In this light, inflammatory biomarkers, such as NLR and PLR, could be used as a preoperative index of AAA sac behavior after EVAR procedures.

## Figures and Tables

**Table 1:** Baseline population study characteristics.

Variables	Population Study (N = 184)
Age (mean ± SD)	75.8 ± 8.3
Male (N,%)	158 (85.9%)
Hypertension (N,%)	155 (84.2%)
Diabetes Mellitus (N,%)	17 (9.2%)
Dyslipidemia (N,%)	128 (69.6%)
CAD (N,%)	52 (28.3%)
PAD (N,%)	16 (8.7%)
AF (N,%)	24 (13%)
CHF (N,%)	45 (24.5%)
Smoking habit (N,%) Active Smoker Former smoker	30 (16.3%) 34 (18.5%)
COPD (N,%)	41 (22.3%)
CKD (N,%)	24 (13%)
Cerebrovascular Disease (N,%)	26 (14.1%)
Malignancy (N,%)	33 (18%)
No. of medication assumed (mean ± SD)	4.9 ± 2.7
Lab Preoperative Variables (mean ± SD)	
Hemoglobin (g/dL) Neutrophil (1000/mL) Lymphocyte (1000/mL) Platelet (1000/mL) NLR PLR Creatinine (mg/dL)	13.9 ± 1.9 5.8 ± 6.2 1.92 ± 1.1 201 ± 61.8 3.6 ± 2.2 135.5 ± 73 0.7 ± 0.9
Drugs Single antiplatelet therapy Dual antiplatelet therapy Direct anticoagulant Statin Vit-K antagonist	102 (55.4%) 34 (18.5%) 42 (22.8%) 148 (80.4%) 15 (8.2%)

Abbreviations: SD: standard deviation; CAD: Coronary Artery Disease; PAD: Peripheral Artery Disease; AF: Atrial fibrillation; CHF: Congestive Heart failure; COPD: Chronic Obstructive Pulmonary Disease; CKD: Chronic Kidney Disease; NLR: Neutrophil-to-Lymphocyte ratio; PLR: Platelet-to-Lymphocyte ratio.

**Table 2:** Procedural and post-operative variables for the population study.

Variables	Population Study (N = 184)
Preoperative AAA diameter (mm) (mean ± SD) Preoperative IMA Patency (N,%)	54 ± 19 95 (51.6%)
Procedural access (N,%) Bilateral percutaneous access Bilateral surgical access Surgical and percutaneous access	48 (26%) 100 (54.4%) 36 (19.6%)
Duration of procedure (min) (mean ± SD)	123 ± 52
Technical success (N,%)	181 (986%)
Post-operative CIN (N,%)	13 (7%)
Hospitalization (days) (mean ± SD)	5.1 ± 2.4
Post-implantation syndrome (N,%)	55 (29.9%)
Inhospital MAE (N,%)	1 (0.5%)
30-day mortality (N,%)	0 (0%)

Abbreviations: AAA: Abdominal Aortic Aneurysm; SD: Standard Deviation; IMA: Inferior Mesenteric Artery; CIN: Contrast-induced Nephropathy; MAE: Major Adverse Events.

**Table 3:** Variable comparison between shrinking AAA and no-shrinking AAA subgroups.

Variables	Shrinking AAA (N = 107)	No Shrinking AAA (N = 77)	p Value
Age (mean ± SD)	74.7 ± 8	77.3 ±8.5	**0.03**
Male (N,%)	93 (86.9%)	65 (84.4%)	0.67
Hypertension (N,%)	91 (85%)	64 (83.1%)	0.83
Diabetes Mellitus (N,%)	11 (10.3%)	6 (7.8%)	0.61
Dyslipidemia (N,%)	75 (70.1%)	53 (68.8%)	0.87
CAD (N,%)	28 (26.1%)	24 (31.2%)	0.5
PAD (N,%)	9 (8.4%)	7 (9.1%)	1
AF (N,%)	12 (11.25)	12 (15.6%)	0.38
CHF (N,%)	26 (24.3%)	19 (24.6%)	1
Smoking habit (N,%) Active Smoker Former smoker	12 (11.2%) 21 (19.6%)	18 (23.4%) 13 (16.9%)	**0.04** 0.7
COPD (N,%)	23 (21.4%)	18 (23.4%)	0.85
CKD (N,%)	14 (13.1%)	10 (13%)	1
Cerebrovascular Disease (N,%)	16 (15%)	10 (12.3%)	0.83
Malignancy (N,%)	20 (18.7%)	13 (16.7%)	0.84
No. of medication assumed (mean ± SD)	5 ± 2.6	4.6 ± 2.6	0.1
Lab Preoperative Variables (mean± SD) Hemoglobin (g/dL) Neutrophil (1000/mL) Lymphocyte (1000/mL) Platelet (1000/mL) NLR PLR Creatinine (mg/dL)	14 ± 2.1 5.3 ± 8.1 2.06 ± 1.3 197.2 ± 61.1 2.77 ± 1.4 114.1 ± 55.7 0.74 ± 1.1	13.7 ± 1.7 5.9 ± 2.8 1.6 ± 0.6 209.1 ± 63.1 4.76 ± 2.5 149.2 ± 74.7 0.7 ± 0.5	0.3 0.53 **0.004** 0.2 **0.0001** **0.0003** 0.7
AAA preoperative diameter (mm) (mean ± SD)	55.7 ± 11.2	53.7 ± 11.2	0.23
Duration of procedure (min) (mean ± SD)	119 ± 43.7	131.1 ± 61.8	0.12
Drugs Single antiplatelet therapy Dual antiplatelet therapy Direct anticoagulant Statin Vit-K antagonist	67 (62.6%) 18 (16.8%) 30 (28%) 91 (85%) 8 (7.5%)	35 (45%) 16 (20.8%) 12 (15.6%) 57 (74%) 7 (9.1%)	0.02 0.56 0.05 0.08 0.8
Technical success (N,%)	106 (99.1%)	75 (97.4%)	0.2
Post-operative CIN (N,%)	8 (7.5%)	5 (6.5%)	1
Hospitalization (days) (mean ± SD)	5.1 ± 2.6	5.2 ± 2.2	1
Post-implantation syndrome (N,%)	34 (31.7%)	21 (27.3%)	0.6
Inhospital MAE (N,%)	0 (0%)	1 (0.9%)	0.4
30-day mortality (N,%) Endoleak	0 (0%) 4 (3.7%)	0 (0%) 9 (11.7%)	1 0.04

Abbreviations: SD: standard deviation; CAD: Coronary Artery Disease; PAD: Peripheral Artery Disease; AF: Atrial fibrillation; CHF: Congestive Heart failure; COPD: Chronic Obstructive Pulmonary Disease; CKD: Chronic Kidney Disease; NLR: Neutrophil-to-Lymphocyte ratio; PLR: Platelet-to-Lymphocyte ratio. Bold *p*-values indicate a statistical significance.

**Table 4:** Stepwise analysis for the predictors of no-shrinking AAA.

Variables for AAA Shrinkage	Hazard Ratio	CI 95%	*p* Value
Age	**2.12**	**1.65–2.73**	**0.04**
Male	1.47	0.72–1.73	0.25
Hypertension	0.90	0.59–2.05	0.1
Diabetes Mellitus	0.81	0.71–1.73	0.3
Dyslipidemia	0.75	0.87–2.18	0.2
CAD	1.48	0.94–2.55	0.4
PAD	1.19	0.66–1.87	0.3
AF	0.78	0.73–1.57	0.4
CHF	1.25	0.63–1.52	0.3
Smoking habit Active Smoker Former smoker	**2.34** 1.52	**1.59–2.81** 0.66–1.58	**0.03** 0.1
COPD	1.23	0.7–1.92	0.2
CKD	1.63	0.81–2.54	0.3
Cerebrovascular Disease	1.35	0.83–2.63	0.2
Malignancy	1.37	0.78–1.83	0.6
N° of medication assumed	1.6	0.88–3.3	0.3
Lab Preoperative Variables Hemoglobin Neutrophil Lymphocyte Platelet NLR PLR Creatinine	1.65 1.19 1.62 1.54 **2.18** **1.89** 1.41	0.75–1.97 0.54–1.67 0.68–2.41 0.71–1.84 **1.57–3.29** **1.42–3.47** 0.671–2.15	0.4 0.2 0.5 0.3 **0.01** **0.03** 0.3
AAA preoperative diameter Preoperative Patent IMA	1.73 1.32	0.74–2.65 0.67–1.85	0.09 0.4
Duration of procedure Endoleak Endoleak with IMA patent	0.78 **1.53** 1.11	0.55–1.41 **1.12–1.94** 0.56–1.75	0.5 **0.03** 0.2
Drugs Single antiplatelet therapy Dual antiplatelet therapy Direct anticoagulant Statin Vit-K antagonist	1.23 1.94 **1.63** 0.97 1.41	0.67–1.65 0.75–2.92 **1.34–2.74** 0.89–1.77 0.72–2.63	0.5 0.1 **0.04** 0.6 0.1
Stepwise Multivariate analysis			
Age	1.48	0.89–1.78	0.4
Active smoker	**2.16**	**1.72–3.04**	**0.04**
NLR	**1.78**	**1.66–2.67**	**0.02**
PLR Endoleak	1.57 1.43	0.91–1.86 0.86–1.56	0.09 0.08
Direct Oral Anticoagulant	1.87	0.77–2.74	0.07
Statin	0.91	0.67–1.89	0.09

Abbreviations: SD: standard deviation; CAD: Coronary Artery Disease; PAD: Peripheral Artery Disease; AF: Atrial fibrillation; CHF: Congestive Heart failure; COPD: Chronic Obstructive Pulmonary Disease; CKD: Chronic Kidney Disease; IMA: Inferior Mesenteric Artery; NLR: Neutrophil-to-Lymphocyte ratio; PLR: Platelet-to-Lymphocyte ratio. Bold *p*-values indicate a statistical significance.

## Data Availability

Data available on request due to privacy restrictions. The data presented in this study are available on request from the corresponding author.
